# Environmental causality calibration: Advancing WLAN RF fingerprinting for precise indoor localization

**DOI:** 10.1371/journal.pone.0297108

**Published:** 2024-02-29

**Authors:** Yufeng Fan, Haotai Sun

**Affiliations:** 1 Shenyang Fire Science and Technology Research Institute of MEM, Shenyang, China; 2 Shenyang Aerospace University, School of Computer Science, Shenyang, China; Polytechnique Montreal (University of Montreal) / Microchip Inc, CANADA

## Abstract

In recent years, considerable and valuable research progress has been made in indoor positioning technologies based on WLAN Radio Frequency (RF) fingerprinting, identifying it as one of the most promising positioning technologies with substantial potential for wider adoption. However, indoor environmental factors significantly influence the propagation of wireless RF signals, resulting in a considerable decrease in positioning accuracy as the indoor environmental conditions vary. Thus, effectively mitigating the impact of indoor environmental factors on WLAN RF fingerprinting-based positioning systems has become a crucial research problem. Currently, there is a dearth of comprehensive research on the influence of indoor climatic factors, particularly the variations in relative humidity, on the propagation of WLAN RF signals within indoor spaces and its consequential impact on positioning accuracy. To address the aforementioned issues, this paper proposes an Adaptive expansion fingerprint database (AeFd) model based on a regression learning algorithm. The AeFd, through the design of a relationship model describing the interaction between fingerprint databases under varying relative humidity, allows the fingerprint database expanded by AeFd to dynamically adapt to the changes in indoor relative humidity. Our experiments show that using the AeFd model with the KNN algorithm, a 5% performance improvement was observed over 10 days and an 8% improvement over 10 months. According to experimental test results, the fingerprint database expansion model AeFd proposed in this paper can effectively expand the fingerprint database under different relative humidity levels, thereby significantly enhancing the positioning performance of the system and improving its stability.

## Introduction

With the rapid development of wireless communication technologies and the widespread prevalence of personal mobile smart devices, the ability for users to obtain accurate indoor location information through their mobile smart devices has become vital across various industries. For instance, in lifestyle services, accurate indoor location information can enhance personalized service delivery [1]. In industrial settings, reliable indoor positioning is essential for efficient workflow and safety [2]. In commercial promotions, location-based strategies can boost customer engagement and sales [3]. In social networking, precise indoor location information can foster a more connected community [4].

Over the years, a variety of indoor positioning techniques and algorithms have been researched, of which those based on infrared, ultrasound [5], RF identification [6, 7], Bluetooth [8], Wi-Fi [9, 10], ultra-wideband [11] and image recognition [12] have gradually become the mainstream techniques. Compared to other indoor positioning technologies, the WLAN RF fingerprinting-based positioning technology, leveraging the widely deployed indoor WLAN network, requires no additional hardware infrastructure. Users only need to use positioning software on their mobile smart devices to satisfy the majority of indoor positioning needs, rendering it one of the indoor positioning methods with the greatest potential for widespread application.

The fundamental principle of WLAN RF fingerprinting-based positioning is to use the attenuation characteristics of RF signals during their propagation through spatial media as spatial position features for positioning. The WLAN RF fingerprinting method is divided into an offline phase and an online phase. In the offline phase, reference points are planned within the positioning area, and RF signals are collected at each access point (AP) to construct a fingerprint database. In the online phase, users send the observed RF fingerprint Received Signal Strength Indicator (RSSI) values received at their location to the fingerprint database, which then estimates the corresponding position coordinates using the positioning algorithm.

According to the principles of WLAN RF fingerprinting-based positioning, in an ideal state, there should exist a one-to-one mapping relationship between RF fingerprints and their corresponding locations. Using fingerprint positioning algorithms would then yield relatively accurate location coordinates. However, wireless RF fingerprint signals used in WLAN RF fingerprinting-based positioning methods are particularly sensitive to environmental factors, which is a commonly accepted understanding during the RF propagation process [13]. Specifically, indoor environmental factors can significantly influence the propagation of wireless RF fingerprint signals, a phenomenon we refer to as “environmental causality.” This “environmental causality” is directly manifested in the fluctuations in wireless RF fingerprint RSSI values in different indoor environments [14, 15], causing the RSSI values observed in real-time during the online phase to potentially deviate from the initial fingerprint database constructed during the training phase. As a result, the positioning accuracy of the system can decrease substantially as the indoor environment dynamically changes.

Previous work has investigated in detail several factors of dynamic indoor environmental changes, clarifying the influences of these factors on the positioning accuracy [16], such as multipath interference, equipment disparity, as well as interference from personnel and building doors and windows. Xie et al. [17] proposed an indoor positioning method based on WLAN RF fingerprinting combines the time difference of arrival (TDOA), which compensated for multipath interference during signal receiving through a rough estimate calculated by the TDoA of WLAN RF fingerprint RSSI signals. Additionally, the PHY channel state information (CSI) with finer granularity was utilized in reference [18]. The CSI outperformed the RSSI in terms of timing and interference immunity, since it could simulate the multipath effect of electromagnetic wave propagation in space. However, at current stage, CSI cannot be directly displayed or acquired on most commercial equipment, which limits its application in the indoor positioning field. Respecting equipment disparity, a linear regression (LR) algorithm was proposed in reference [19] to eliminate the equipment disparity in the WLAN RF fingerprint positioning system, and the RSSI variation resulting from hardware disparity was modeled by using LR equation. In reference [20], the equipment disparity problem was addressed by adopting a hierarchical Bayesian model combined with the conditional independent parameters related to each transmitter and receiver. Modeling was attempted in reference [21] by considering multiple indoor environmental factors. Additional RFID and environmental sensors were used to confirm whether indoor personnel were walking or whether doors and windows were closed, and the WLAN RF fingerprint database was collected according to different environments. However, this method was not applicable on a large scale due to the need for additional infrastructure. The aforementioned work has alleviated the influences of environmental factors on the indoor positioning performance to some extents. Nevertheless, the impact of indoor climatic factor variations on the WLAN RF propagation in indoor spaces has received inadequate attention. There especially lacks in-depth research concerning the influence of changes in indoor relative humidity (RH) on the position estimation accuracy in the final online phase.

**Fig 1:**
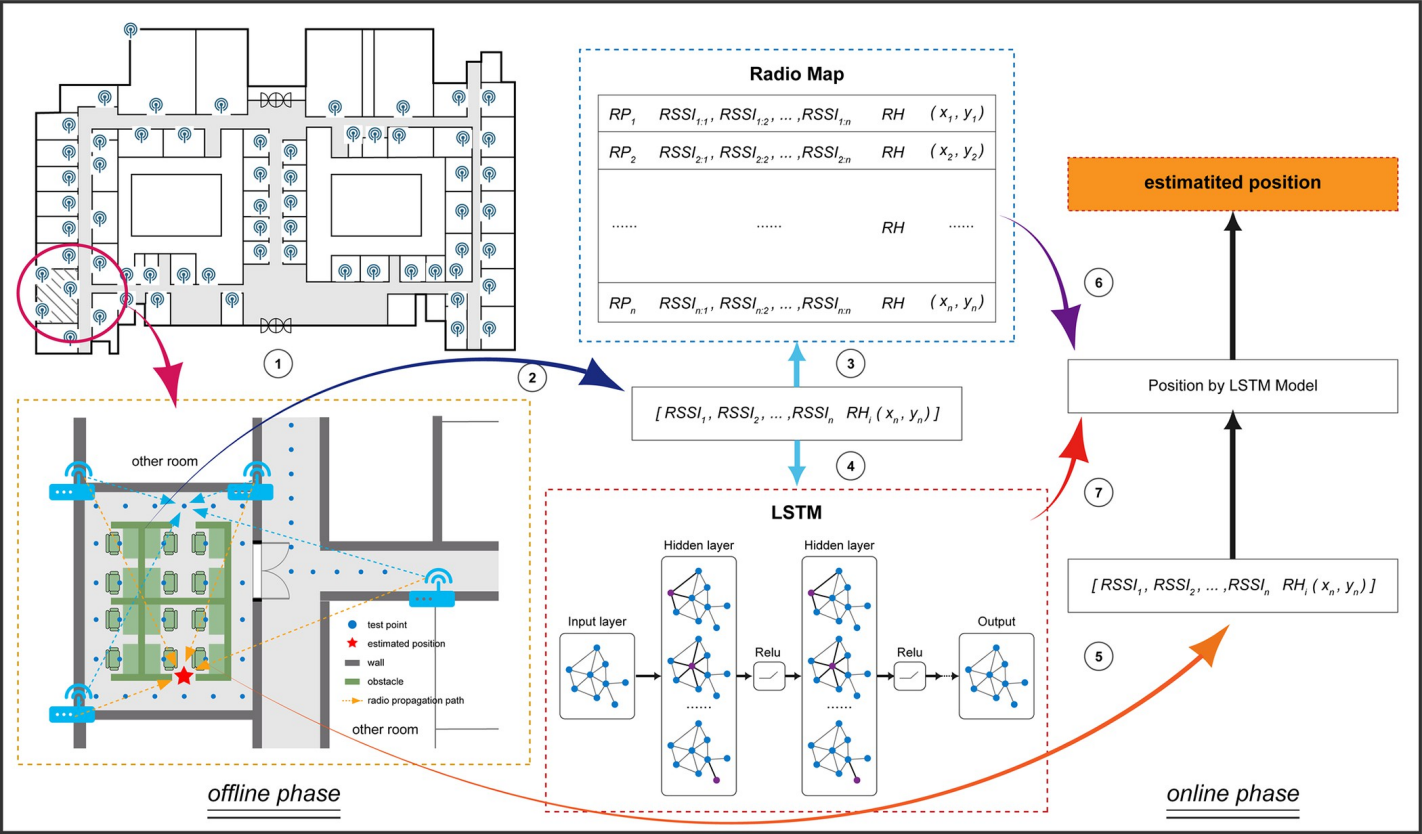
AeFd workflow overview.

To address the aforementioned problem, this paper first conducts in-depth research and analysis on the implicit impact of indoor climatic factors on positioning performance fluctuations, and explicitly uncovers a factor that has not been given sufficient attention or even discovered before–indoor relative humidity–which nonetheless significantly influences the accuracy of fingerprint matching positioning. Secondly, to mitigate the impact of indoor relative humidity, we propose a model named Adaptive Expansion Fingerprint Database (AeFd). This model is based on regression learning algorithms, requiring no additional hardware investments or human resources. It realizes the adaptive expansion of the RF fingerprint database collected under known indoor relative humidity conditions in response to varying indoor relative humidity environments. Fig 1 illustrates the workflow of the method proposed in this study.

In our proposed method, we first establish reference points within the positioning area and collect Radio Frequency (RF) fingerprint Received Signal Strength Indicator (RSSI) values from multiple Access Points (APs) to construct a primary fingerprint database (Step 1). Further RF fingerprint RSSI values are gathered for database expansion (Step 2). We then employ the Adaptive Expansion Fingerprint Database (AeFd) model for automatic and continuous database expansion, enabling dynamic adaptation to indoor humidity changes (Step 3). Simultaneously, a positioning algorithm is developed to estimate user locations using the expanded database (Step 4). In practical application, the user’s device collects real-time WLAN RF fingerprint RSSI values (Step 5), which are input into the positioning algorithm, aligning with the current indoor humidity level (Step 6). Finally, the user’s location coordinates are estimated and output using the positioning algorithm and the expanded database (Step 7).

The main contributions of this paper include the following points:

1. This paper deeply explores and analyzes the potential influence of indoor climate factors on positioning performance fluctuations. We clearly reveal an aspect that has not been sufficiently noticed or even discovered before—indoor relative humidity. This factor significantly impacts the precision of WLAN RF fingerprint-based positioning. Furthermore, we found that due to the disregard of the indoor relative humidity factor, the fingerprint database cannot adapt to the dynamic changes in indoor relative humidity. This results in significant deviations between fingerprint data and actual values, further reducing the positioning accuracy in the online stage.
2. This paper designs and proposes for the first time an Adaptive Expansion Model for WLAN RF fingerprinting—AeFd. By utilizing regression learning algorithms, the model forms a relationship between fingerprint databases under different indoor relative humidity conditions and adaptively expands the entire fingerprint database. The model proposed in this paper requires no additional hardware deployment or labor cost. It achieves continuous and adaptive expansion of the WLAN RF fingerprint database, enabling a statically constructed fingerprint database to dynamically adapt to the impact of indoor environmental relative humidity changes on the fingerprint database during long-term operation.
3. Experiments were conducted in a real-world environment to verify the proposed method. The results show that the AeFd adaptive expansion model proposed in this paper can effectively adapt to the dynamic fluctuations of indoor relative humidity. It also maintains accurate and stable positioning performance based on WLAN RF fingerprint positioning methods.

## Scrutinizing challenges and their repercussions

### RSSI fluctuation problem

To delve deeper into the impact of indoor climatic factors on WLAN RF fingerprint RSSI values, we deployed a positioning system prototype in a real environment. This prototype system, based on a hybrid dual RF fingerprint database, operated for over 10 months, with experimental testing days exceeding 100. However, during the long-term tracking experiment, we found that the positioning accuracy of the built fingerprint database fluctuated considerably over a long time period. This leads us to question whether there are other overlooked factors influencing the propagation of RSSI indoors.

**Fig 2:**
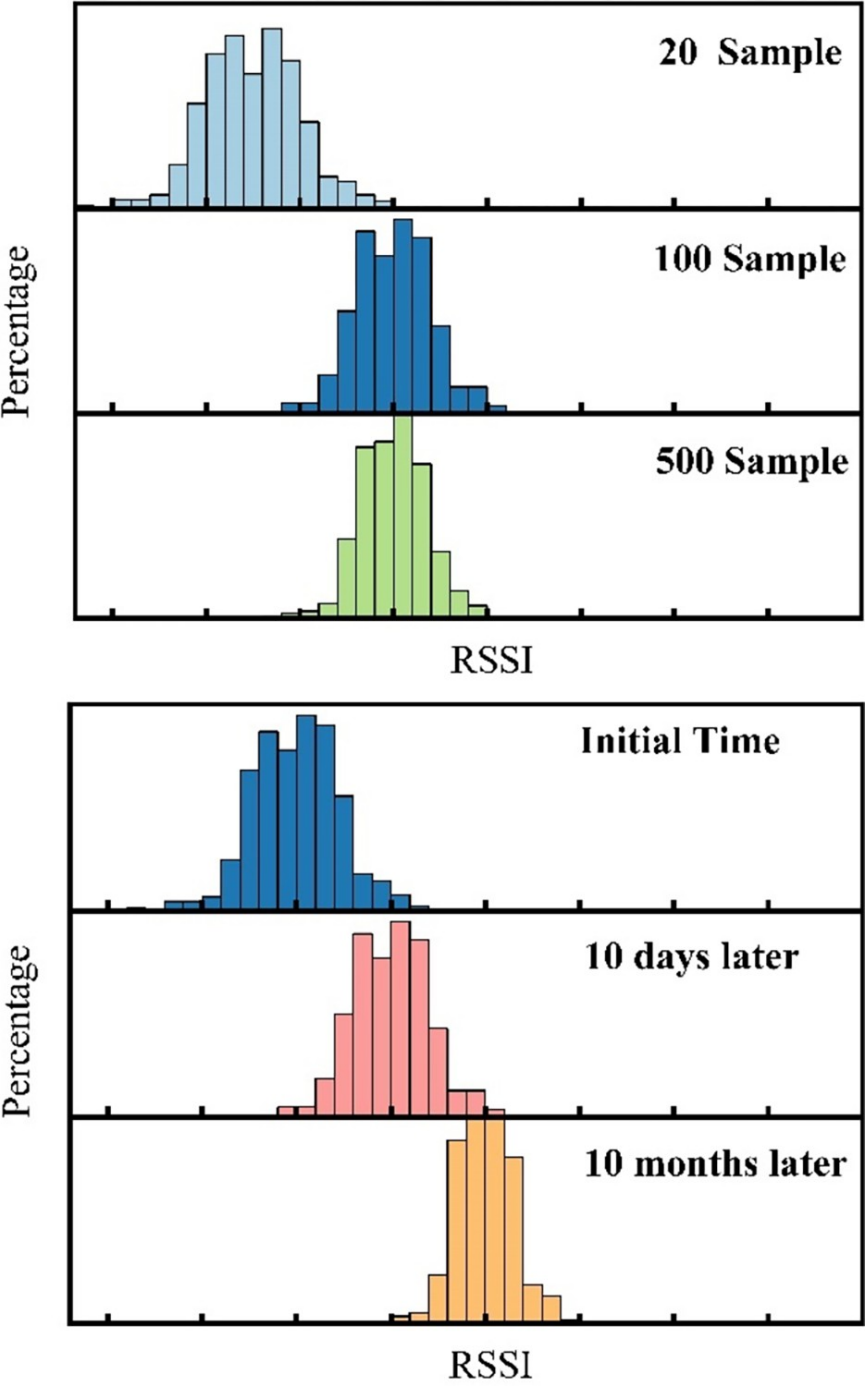
Periodic fluctuations of RSSI over time. (a) RSSI distributions formed by different measurement times of collected samples (b) Deviation of RSSI fingerprint from the initial database significantly increases over time.

To investigate this, we designed an experiment to longitudinally monitor changes in RSSI data and analyze the causes of positioning accuracy fluctuation in detail. Specifically, under the premise of real-time recording of indoor temperature and relative humidity, by continuously recording the RSSI values from the same AP at a fixed location, we obtained some significant findings as shown in Fig 2. These include the following aspects:

1. Fig 2(A) shows the distribution of RSSI values sampled 20, 100, and 500 times at a fixed location. It can be observed that the RSSI values obtained from only 20 samples are not sufficient to characterize the RSSI distribution at a specific location. However, when the number of samples increases to 100 and 500, the overall RSSI distribution tends to stabilize, accurately reflecting the RSSI distribution at the specific location. This explains why, during the fingerprint collection process, it is necessary to stay at a reference point for a sufficient length of time and take multiple samples.
2. Fig 2(B) presents the distribution of 100 RSSI samples at a fixed location under different sampling times. The results indicate that, compared to the RSSI of the initial sampling period, significant offsets occur in the RSSI values over time. Within a relatively short period (for example, 10 days), the offset of RSSI values is relatively small. However, over a longer period (for example, 10 months), the offset of RSSI values notably increases. This explains why the fingerprint database initially built gradually deviates from the true value in the positioning system deployed over the long term, which may eventually lead to increased positioning errors in the online phase.

Based on the analysis of foregoing experimental results, the instantaneous jump of RSSI was caused by indoor personnel movement or potential interference sources. Such RSSI jump can be alleviated through multiple repeated samplings in different directions by staying at each sampling point for a sufficiently long time [22], or the jump data can be eliminated via filtering algorithms [23]. However, the RSSI value fluctuated periodically over a long time, which was found to be caused by indoor climatic changes. After all, the propagation of electromagnetic waves in the space medium would change with changes in climatic factors. For instance, the rain attenuation of electromagnetic waves was attributed to the RF absorption and attenuation by the raindrops in the atmosphere, which ultimately affected the communication performance [24].

## Influence of temperature on RSSI

**Fig 3:**
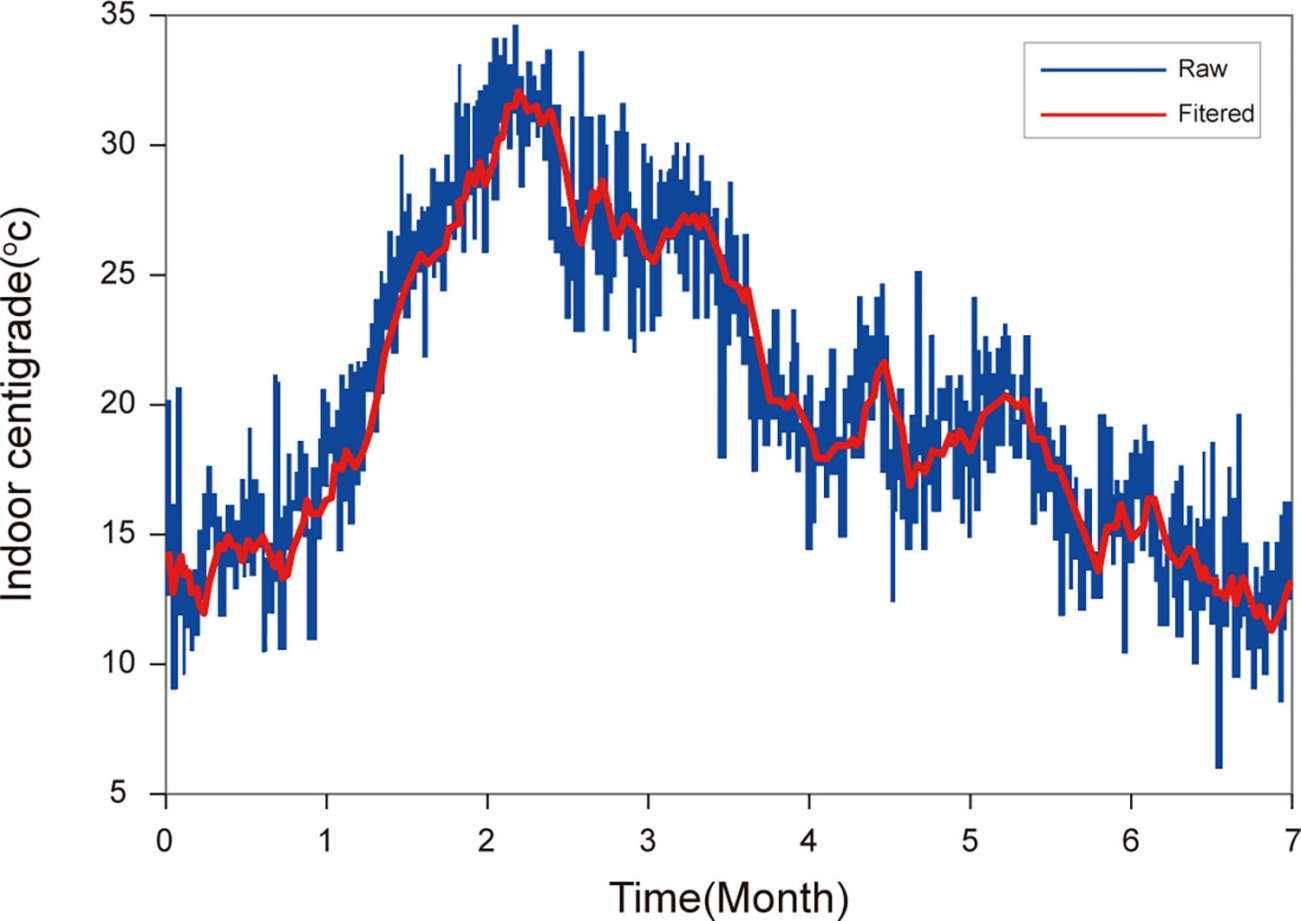
Indoor temperature variation curves. The experiment started in April and ended in November.

The experimental design in this section considers tracking and collecting indoor temperature data over different time periods, dates, and months. The experiment started in April and ended in November. As shown in Fig 3, it displays the variations in indoor temperature. The observation results indicate that, in the indoor environment, due to the widespread use of facilities such as air conditioning, heating, and building insulation materials, the overall fluctuation range of indoor temperature is predominantly between 15°C and 30°C.

In order to visually analyze the impact of temperature on RSSI, this section designs a visualization experiment environment. To exclude the interference of other indoor factors on the experimental results, all experiments are carried out in an environment where the indoor relative humidity is kept constant and there is no human movement. The experimental steps are as follows: A uniform receiving apparatus, specifically a Huawei Mate30 Pro smartphone, was employed to record the signal strength emanating from an AP positioned in a fixed direction. To mitigate the interference potentially introduced by diverse equipment manufacturers, systematic measurements of RSSI were conducted across AP devices sourced from ten distinct manufacturers.

**Fig 4:**
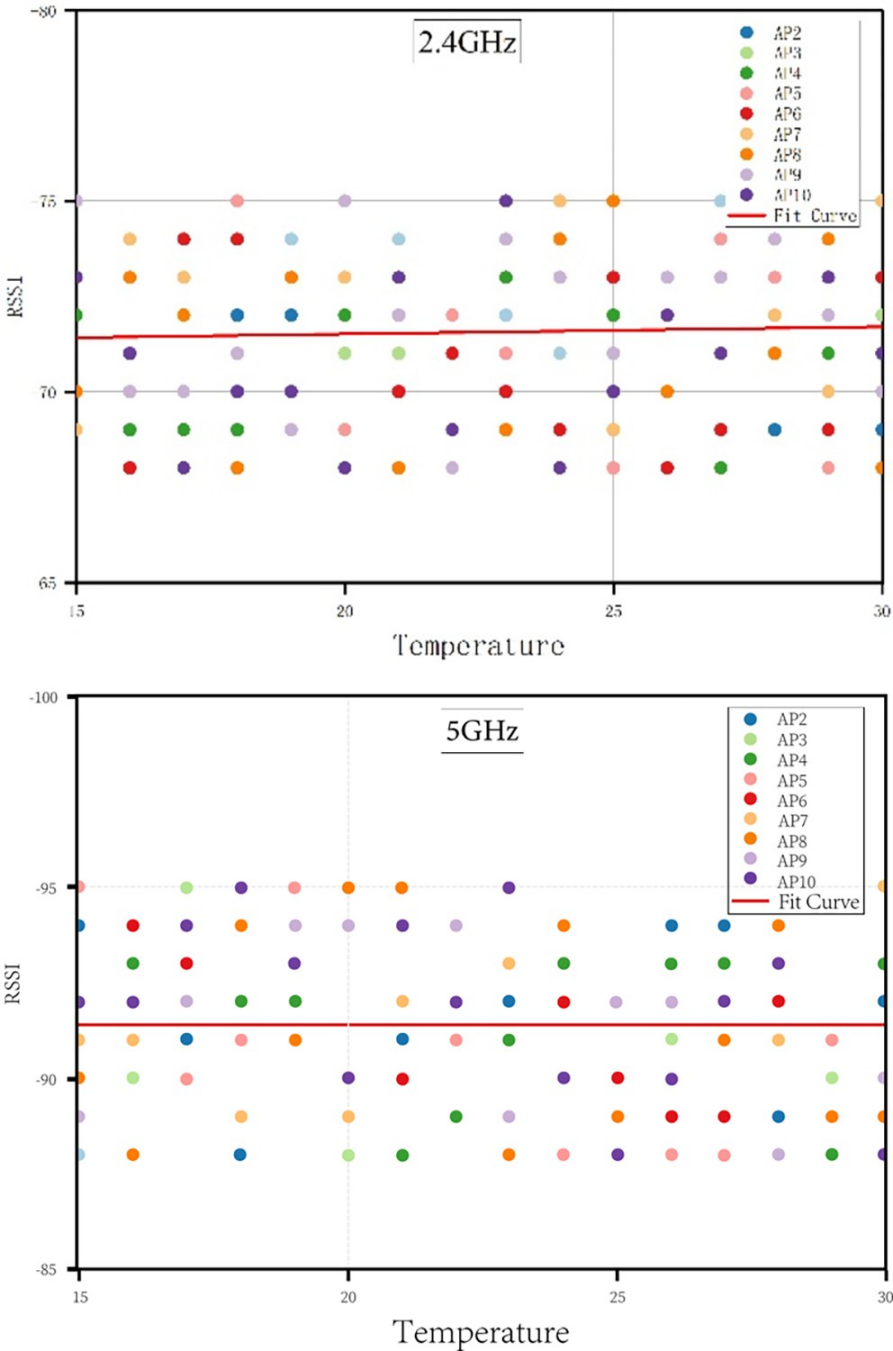
Influence of indoor temperature changes on 2.4GHz and 5GHz RSSI values.

Fig 4 presents the mean RSSI values for ten AP devices measured at a fixed indoor location under constant relative humidity conditions at temperatures of 15°C, 20°C, 25°C, and 30°C. Each AP device is represented by a distinct color in the illustration. Therefore, we averaged the RSSI values for both 2.4G and 5G frequencies. Let’s denote the mean RSSI value at 2.4G and 5G as *RSSI*_*avg*_. Then, we applied the least squares method to fit *RSSI*_*avg*_ under varying indoor temperature conditions. The fitting equation can be represented as: *RSSI*_*avg*_ = a * T + b. where T represents the indoor temperature, and a and b are the coefficients determined by the least squares fitting method. The red curve in Fig 4 represents the fitted curve of *RSSI*_*avg*_ values. As observed from the curve, despite minor fluctuations in the *RSSI*_*avg*_ for 2.4G and 5G, there isn’t a discernible trend of change. This aligns with the experimental results conducted in outdoor environments as documented in references [25–27]. Therefore, we have substantial evidence to conclude that the RSSI values of 2.4G and 5G are not significantly affected by indoor temperature variations within the range of 15–30°C.

### Influence of humidity on RSSI

**Fig 5:**
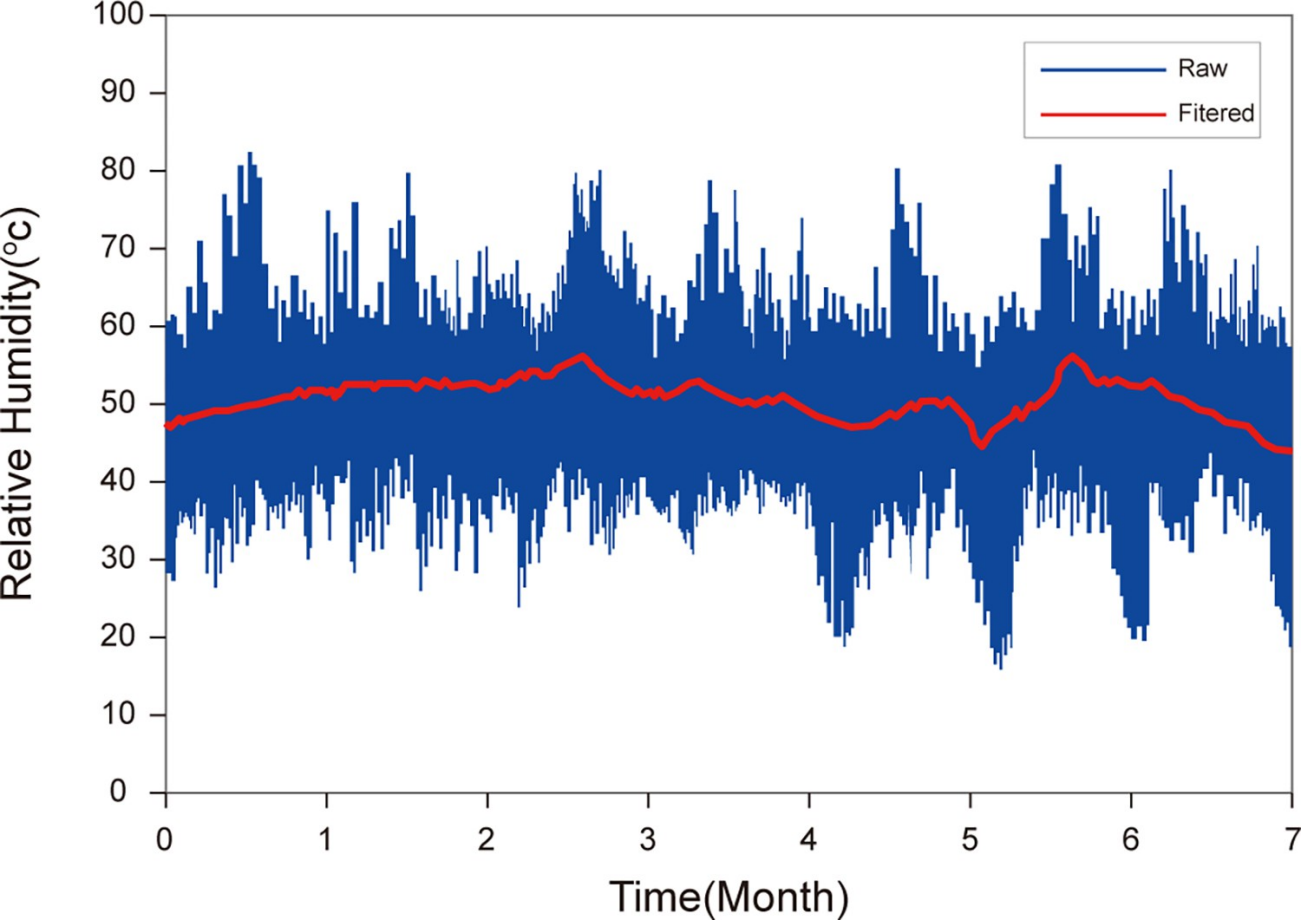
Indoor RH variation curves.

To verify the influence of indoor RH on the WLAN RF fingerprint RSSI, the indoor RH data were collected multiple times at different times in different seasons under different weather conditions. Fig 5 illustrates the indoor RH variations. Statistical analysis revealed that the RH in the indoor working and living environments fluctuated drastically with the alternation of seasons and weather. For instance, rain, dense fog and other weather would all greatly affect the RH indoors. Hence, the indoor RH changed drastically within the range of 20–80% in general.

For intuitive presentation of the indoor RH influence on RSSI, an experimental context under visual condition was designed in this section, where the experimental signal path was unobstructed. That is, the RF signals between the experimental terminals and the APs were visually transmitted. The specific experimental steps were as follows: the same APs (Xiaomi MI 4C) from the same directions were measured with the same receiving devices (Huawei Mate30 Pro smartphone). The RH in the experimental environment was controlled by a large-capacity air humidifier. When the temperature remained constant, ten points in the experimental site were randomly selected for measurement with a hygrometer. The randomly measured values were averaged and used as the indoor relative at that time. The RH in the experimental site was controlled by constantly adjusting the humidifier, so that the expected humidity could be attained.

**Fig 6:**
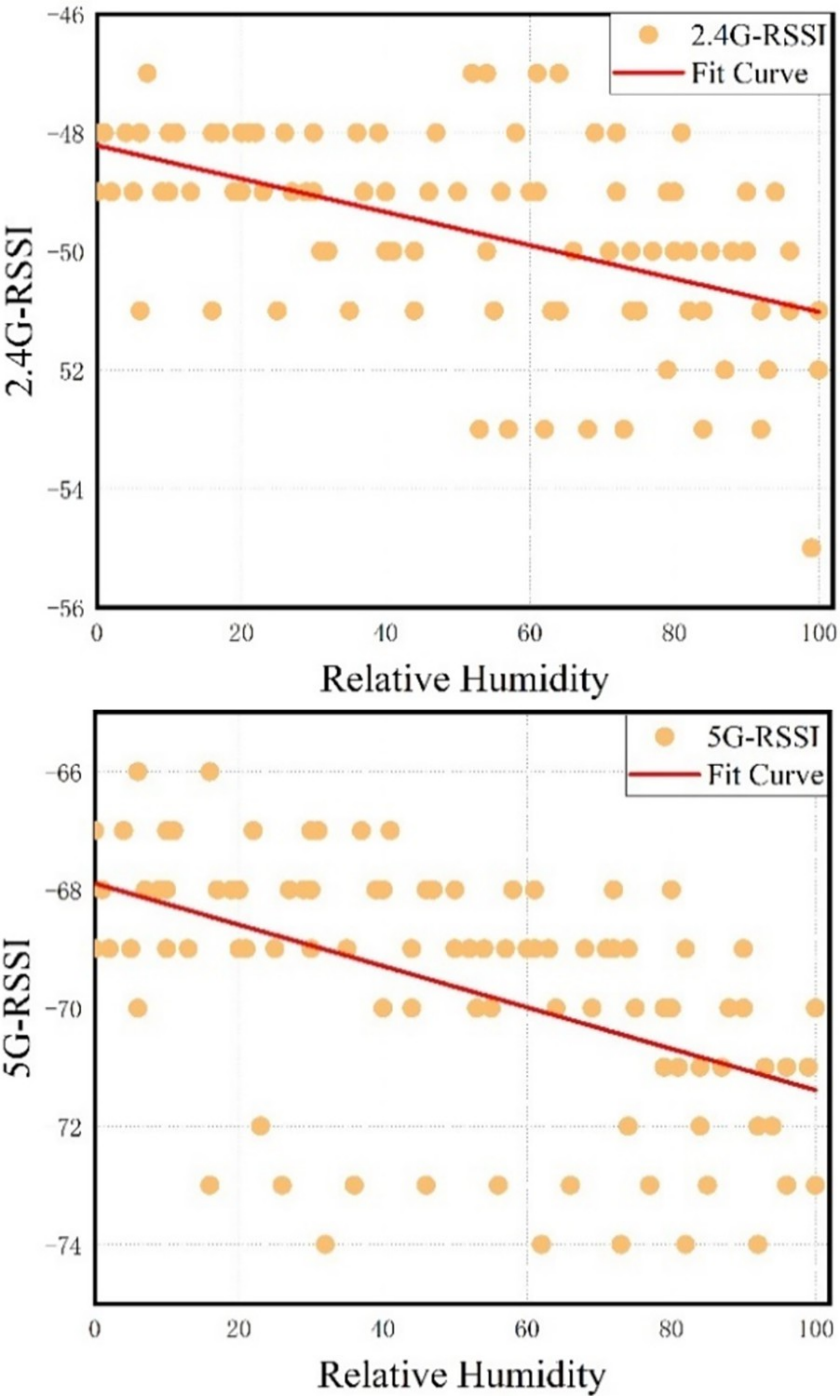
Influence of indoor relative humidity variations on 2.4GHz and 5GHz RSSI values.

Fig 6 depicts the variation intervals of RSSI distribution from the same AP at the same reference position under the indoor RH condition of 0–100%. To avoid the interference of other factors with this experiment, investigation was performed on the premise of keeping no interference indoors. As is clear from Fig 6, an obvious correlation was present between RH and signal strength. Generally, the RSSI value decreased when the RH increased, and vice versa.

Meanwhile, the above results well explain the problem found in Section 2.1. That is, the change in the indoor RH caused fluctuation of the WLAN RF fingerprint RSSI, thereby leading to evident deviation or volatility in the periodic positioning with the built fingerprint database over a long time. Precisely due to negligence of the correlation between the RSSI fingerprint feature at reference point and the indoor RH factor, the statically constructed fingerprint database became defective. In other words, the fingerprint database failed to cover the fingerprint features under all the indoor RH conditions. As a result, position estimation was implemented in the online phase by matching the RF fingerprint RSSI at a 40% indoor RH with the fingerprint database at an 80% indoor RH, ultimately resulting in unstable positioning performance.

### Correlation between humidity and RSSI

To quantitatively analyze the correlation between RF fingerprint RSSI and indoor RH, the Pearson correlation coefficient was employed in this section to perform relevant correlation analysis. This method reflects the relationship between feature and response, which can well measure the linear correlation between variables [28].

The value interval of the Pearson correlation coefficient result was [–1,1]. When the correlation coefficient was -1, it indicated a strong negative linear correlation; when the correlation coefficient was +1, it indicated a strong positive linear correlation; and when the correlation coefficient was 0, it indicated irrelevance. The computational formula is as follows:

$$Correlation=(\frac{Cov(RH,RSSI)}{\sqrt{Var[RH]Var[RSSI]}})$$ (1)

where *RH* denotes the indoor RH, *Cov*(*RH*,*RSSI*) stands for the covariance between *RH* and *RSSI*, *Var*[*RH*] is the variance of *RH*, and *Var*[*RSSI*] the variance of *RSSI*.

The correlation coefficients between RH and 2.4G-RSSI and 5G-RSSI are presented in Table 1. As can be seen from Table 1, RH shows a strong negative correlation with both 2.4G-RSSI and 5G-RSSI. Specifically, the correlation coefficient between RH and 2.4G-RSSI is -0.784, while that between relative humidity and 5G-RSSI is -0.813. This indicates that the fluctuation in RSSI can largely be explained by changes in indoor relative humidity. Furthermore, Table 1 reveals differences in correlation coefficients between 2.4G-RSSI and 5G-RSSI. This is due to the different attenuation characteristics of RF fingerprint RSSI at different frequencies in indoor environments with different relative humidities.

**Table 1:** Correlation coefficient between RH and RF fingerprint RSSI.

**Samples**	**2.4G-RSSI**	**5G-RSSI**
**RH range**	0–100%	0–100%
**Correlation coefficient**	-0.784	-0.813

## The AeFd methodology

### Data collection

The collection of data for model training employed a manual approach. The area of the data collection site was 20 square meters, utilizing 4 Xiaomi MI 4C AP devices. A total of 9 reference points were established with a distance of 2 meters between each point. The experiment spanned an indoor relative humidity range from 0% to 100%. The indoor relative humidity was controlled and maintained by six Xiaomi humidifiers. At each reference point, 20 pieces of RF fingerprint RSSI data were collected in different directions.

The WLAN RF fingerprint-based database is as follows:

$$D=[\begin{array}{ccccc} x_{1} & y_{1} & RSSI_{1}^{1} & \ldots & RSSI_{1}^{K}\\ x_{2} & y_{2} & RSSI_{2}^{1} & \ldots & RSSI_{2}^{K}\\ & & & \vdots & \\ x_{n} & y_{n} & RSSI_{n}^{1} & \cdots & RSSI_{n}^{K} \end{array}]$$ (2)

Given the necessity to consider the indoor RH factor into the AeFd model during collection of training data, on the basis of Formula 3, Wherein, *x* represents the x-axis coordinate on the indoor floor plan, *y* signifies the y-axis coordinate on the same floor plan, and RSSI denotes the RSSI value received at location (*x*,*y*). Furthermore, n indicates the number of indoor traversals conducted, and m represents the quantity of Access Points (APs) received at that specific point. the initial fingerprint database $D_{RH}^{0}$ to be expanded by AeFd in this study was formed by collecting and recording the indoor RH value at that time as the indoor humidity label.

$$D_{RH}^{0}=[\begin{array}{cccccc} x_{1} & y_{1} & RH & RSSI_{1}^{1} & \cdots & RSSI_{1}^{K}\\ x_{2} & y_{2} & RH & RSSI_{2}^{1} & \cdots & RSSI_{2}^{K}\\ & & & \vdots & & \\ x_{n} & y_{n} & RH & RSSI_{n}^{1} & \cdots & RSSI_{n}^{K} \end{array}]$$ (3)

where *RH* denotes the indoor relative humidity, *x* and *y* respectively stand for the *x* and *y* coordinates of indoor floor plan, *RSSI* indicates the RSSI value of WLAN RF fingerprint at the coordinate point (*x*,*y*), *n* denotes the number of reference points, *K* denotes the number of APs received at the reference point, and $D_{RH}^{0}$ refers to the initial fingerprint database to be expanded in this Chapter.

### AeFd expansion model

The typical linear regression model [27] is *Y* = *Xβ*+*ε*, where the least squares estimate of parameter *β* is:

$$\hat{\beta }={\left(X^{T}X\right)}^{-1}X^{T}Y$$ (4)

The problem in this chapter is to explore the correlation between two sets of multiple variables, and to predict one set of variables (expanded fingerprint database ${\hat{D}}_{i,j}(RH)$) using another set of variables (initial fingerprint database *D*_*i*,*j*_(*RH*_0_)). Accordingly, it can be seen from Formula 4 that when (*X*^*T*^*X*) was irreversible or approximately irreversible, $MSE(\hat{\beta })$ would be considerably large, and the estimated value $\hat{\beta }$ would be very unstable. The Mean Squared Error is a common measure used in statistical models to quantify the difference between the predicted and actual values. In this case, the typical least squares regression model would produce unstable estimated coefficients, leading to unstable prediction results. Hence, the partial least squares (PLS) regression was employed herein, which could still obtain a stable and correct prediction function model when collinearity was present between the observed variables [29].

Assuming there were *p* dependent variables $RSSI_{RH_{0}}^{1}$, $RSSI_{RH_{0}}^{2}$,…, $RSSI_{RH_{0}}^{n}$ under the initial indoor RH condition *RH*_0_, as well as *p* independent variables $RSSI_{RH}^{1}$ under the predicted RH condition *RH*. The normalized measurement matrices for the dependent and independent variables are respectively denoted as:

$$F_{0}=[\begin{array}{ccc} RSSI_{RH_{0}}^{11} & \cdots & RSSI_{RH_{0}}^{1p}\\ \vdots & \ddots & \vdots \\ RSSI_{RH_{0}}^{n1} & \cdots & RSSI_{RH_{0}}^{np} \end{array}]$$ (5)

$$E_{0}=[\begin{array}{ccc} RSSI_{RH}^{11} & \cdots & RSSI_{RH}^{1p}\\ \vdots & \ddots & \vdots \\ RSSI_{RH}^{n1} & \cdots & RSSI_{RH}^{np} \end{array}]$$ (6)

The PLS modeling steps are as follows:

(1) The first principal components of dependent variable *F*_0_ and independent variable *E*_0_ were separately extracted to maximize their correlation.

Assuming the first principal components *t*_1_ and *u*_1_ were separately extracted from two sets of variables, then *t*_1_ was the linear combination $t_{1}=w_{11}RSSI_{RH_{0}}^{1}+\ldots +w_{1p}RSSI_{RH_{0}}^{p}$ of independent variable $X={\left(RSSI_{RH_{0}}^{1},\ldots ,RSSI_{RH_{0}}^{p}\right)}^{T}$, and *u*_1_ was the linear combination $u_{1}=v_{11}RSSI_{RH}^{1}+\ldots +v_{1p}RSSI_{RH}^{p}$ of dependent variable $Y={\left(RSSI_{RH}^{1},\ldots ,RSSI_{RH}^{p}\right)}^{T}$.

To holistically investigate the correlation between the two sets of variables, the following requirements were imposed:

- Variation information of *t*_1_ and *u*_1_ should be extracted from the respective variable sets as much as possible.
- The correlation between *t*_1_ and *u*_1_ should reach the maximum.

Through the normalized measurement matrices *E*_0_ and *F*_0_ of the two sets of independent and dependent variables, the score vectors for the first pair of principal components were separately calculated, which were denoted as ${\hat{t}}_{1}$ and ${\hat{u}}_{1}$:

$${\hat{t}}_{1}=E_{0}w_{1}=[\begin{array}{ccc} RSSI_{RH}^{11} & \cdots & RSSI_{RH}^{1p}\\ \vdots & \ddots & \vdots \\ RSSI_{RH}^{n1} & \cdots & RSSI_{RH}^{np} \end{array}][\begin{array}{c} w_{11}\\ \vdots \\ w_{1p} \end{array}]=[\begin{array}{c} t_{11}\\ \vdots \\ t_{1p} \end{array}]$$ (7)

$${\hat{u}}_{1}=F_{0}V_{1}=[\begin{array}{ccc} RSSI_{RH_{0}}^{11} & \cdots & RSSI_{RH_{0}}^{1p}\\ \vdots & \ddots & \vdots \\ RSSI_{RH_{0}}^{n1} & \cdots & RSSI_{RH_{0}}^{np} \end{array}][\begin{array}{c} v_{11}\\ \vdots \\ v_{1p} \end{array}]=[\begin{array}{c} u_{11}\\ \vdots \\ u_{1p} \end{array}]$$ (8)

The inner product of ${\hat{t}}_{1}$ and ${\hat{u}}_{1}$ could be used to calculate the covariance *Cov*(*t*_1_,*u*_1_) of the first pair of components. Hence, the above two requirements could be transformed into a conditional value problem, as shown in the formula below:

$$\{\begin{array}{l} \langle {\hat{t}}_{1},{\hat{u}}_{1}\rangle =\langle E_{0}w_{1},F_{0}v_{1}\rangle =w_{1}^{T}E_{0}^{T}F_{0}v_{1}\Rightarrow Max\\ w_{1}^{T}={\left\|w_{1}\right\|}^{2}=1,v_{1}^{T}v_{1}={\left\|v_{1}\right\|}^{2}=1 \end{array}$$ (9)

By Lagrangian multiplier method, the problem was transformed into solving the unit vectors *w*_1_ and *v*_1_, and making $\theta_{1}=w_{1}^{T}E_{0}^{T}F_{0}v_{1}\Rightarrow Max$. Thus, it was only necessary to calculate the eigenvalue and eigenvector of the matrix $M=E_{0}^{T}F_{0}F_{0}^{T}E_{0}$, and make the maximum eigenvalue of *M* be $\theta_{1}^{2}$. The corresponding unit vector was precisely the solution *w*_1_ to be sought. Meanwhile, *v*_1_ could be calculated by $v_{1}=\frac{1}{\theta_{1}}F_{0}^{T}E_{0}w_{1}$.

(2) Regression model was built by regressing $RSSI_{RH}^{1}$,…, $RSSI_{RH}^{p}$ against *t*_1_ and by regressing $RSSI_{RH_{0}}^{1}$,…, $RSSI_{RH_{0}}^{p}$ against *u*_1_ as

$$\{\begin{array}{l} E_{0}={\hat{t}}_{1}\alpha_{1}^{T}+E_{1}\\ F_{0}={\hat{t}}_{1}\beta_{1}^{T}+F_{1} \end{array}$$ (10)

In the above formula, *α*_1_ = (*α*_11_,…,*α*_1*p*_)^*T*^ and *β*_1_ = (*β*_11_,…,*β*_1*p*_)^*T*^ respectively denote the parameter vectors in the many-to-one regression model, and *E*_1_, *F*_1_ are the residual matrices. Then, the least square estimates of regression coefficient vectors are:

$$\{\begin{array}{l} \alpha_{1}=E_{0}^{T}{\hat{t}}_{1}/{\left\|{\hat{t}}_{1}\right\|}^{2}\\ \beta_{1}=F_{0}^{T}{\hat{t}}_{1}/{\left\|{\hat{t}}_{1}\right\|}^{2} \end{array}$$ (11)

where *α*_1_ and *β*_1_ were called the model effect loads.

(3) The above steps were repeated by replacing *E*_0_ and *F*_0_ with the residual matrices *E*_1_ and *F*_1_.

Suppose ${\hat{E}}_{0}={\hat{t}}_{1}\alpha_{1}^{T}$ and ${\hat{F}}_{0}={\hat{t}}_{1}\beta_{1}^{T}$, then the residual matrices were $E_{1}=E_{0}-{\hat{E}}_{0}$ and $F_{1}=F_{0}-{\hat{F}}_{0}$. If the original in the residual matrix *F*_1_ was approximately equal to 0, it indicated that the regression analysis establishing the first principal component already satisfied the requirements, so the component extraction could be terminated. Otherwise, the above steps were repeated by replacing *E*_0_ and *F*_0_ with the residual matrices *E*_1_ and *F*_1_.

(4) Next, the second principal components were separately extracted to maximize their correlation.

By substituting $t_{k}=w_{k}^{*}RSSI_{RH_{0}}^{1}+\ldots +w_{k}^{*}RSSI_{RH_{0}}^{p}(k=1,2,\ldots ,r)$ into *Y* = *t*_1_*β*_1_+…+*t*_*r*_*β*_*r*_, the PLS regression method of *p* dependent variables was obtained:

$$RSSI_{RH}^{j}=\alpha_{j1}RSSI_{RH_{0}}^{1}+\ldots +\alpha_{jp}RSSI_{RH_{0}}^{p}(j=1,2,\ldots ,p)$$ (12)

Accordingly, the prediction model could be obtained as shown in the formula below:

$${\hat{RSSI}}_{RH}^{j}=XB=XW{\left(P^{T}W\right)}^{-1}\hat{C}$$ (13)

where $\hat{RSSI_{RH}^{j}}$ denotes the predicted value and $B=W{\left(P^{T}W\right)}^{-1}\hat{C}$ is the regression coefficient.

### Model training

By inputting the fingerprint data trained at different indoor RHs collected in section 3.1 into the AeFd model, the regression coefficient of adaptive expansion model was determined via the fingerprint database training models under different indoor RH conditions. Meanwhile, the RSSI fingerprint values at different RHs were obtained, as shown in Formula 14:

$$\hat{RSSI_{ij}}(RH)=f(RSSI_{ij}(RH_{0}))(RH\neq RH_{0})$$ (14)

where *RSSI*_*ij*_(*RH*_0_) represents the RSSI value of *j*th AP at the *i*th position under a *RH*_0_ condition, *f*(∙) stands for the regression coefficient and $\hat{RSSI_{ij}}(RH)$ is the RSSI value of *j*th AP at the *i*th position under a *RH* condition, where *RH*≠*RH*_0_.

### Fingerprint database expansion

After completion of the AeFd model training, the regression coefficients were determined. In this section, fingerprint data collected at a series of reference points were used to expand and complement the fingerprint database under different RH conditions. Noteworthy was that the prerequisite for the adaptive expansion of initial fingerprint database was: at least a set of RSSI values were collected at each reference point under a certain RH condition, eventually forming the fingerprint data at the corresponding reference point under the corresponding RH condition. Table 2 details the algorithm for fingerprint database expansion.

**Table 2:** AeFd adaptive expansion algorithm.

**Algorithm1: AeFd adaptive expansion model**
**Input:** The fingerprint data measured at a series of reference points *l*_*i*_ under an indoor *RH*_0_ condition at the initial moment using the fingerprint database *D*_*ij*_(*RH*_0_). **Output:** The fingerprint database $\hat{D_{ij}}(RH)$ to be expanded under different indoor *RH*(*RH*≠*RH*_0_) conditions after updating and expansion via the AeFd model. 1: for each position *l*_*i*_, do 2: for each AP *j*, do 3: determine the model regression coefficient according to the *RSSI*_*ij*_(*RH*_0_) calculation Formula 13 4: expand *D*_*ij*_(*RH*_0_) to $\hat{D_{ij}}(RH)$ under different *RH* conditions according to the Formula 14 5: end for 6: end for

## Experimental results

### Experimental setup

**Fig 7:**
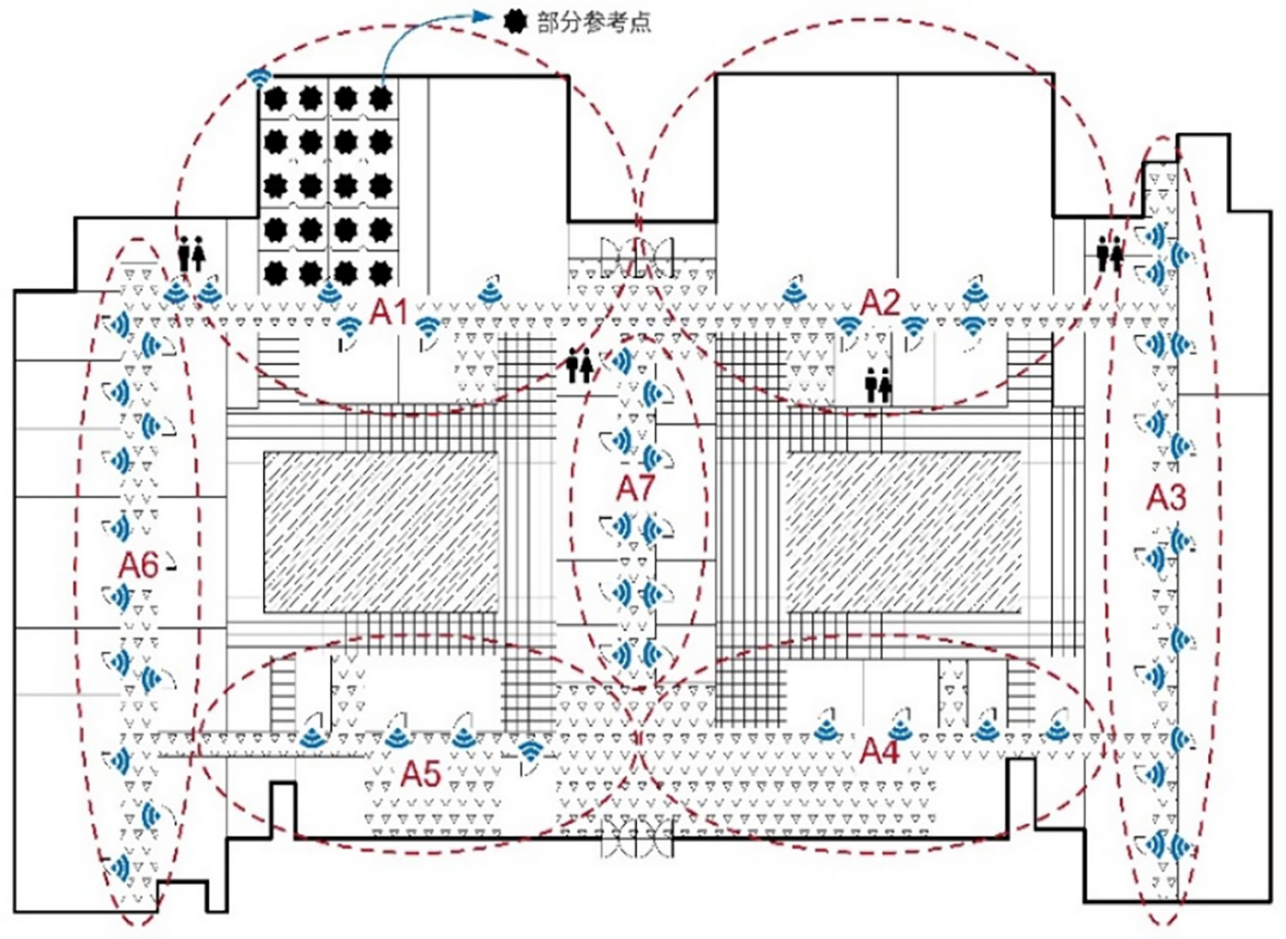
Floor plan of indoor environment.

To validate the positioning performance of the system prototype, this section designs the experimental scenario as depicted in Fig 7. The experimental scenario is within a teaching building covering an area of over 2600 square meters. The test area includes several corridors and rooms, with the rooms incorporating typical indoor settings such as offices, classrooms, and laboratories. Two hundred reference points were arranged at a 1*m*×1*m* density at the experimental site, and the RF fingerprint data at all reference points were collected within the positioning area. The data collection was performed at different times in different seasons under different weather and humidity conditions, and the indoor RHs collected each time were recorded in detail. 20 fingerprint data were collected at each sampling point. After the collection of data, the AeFd model was used to expand the fingerprint database, so that the database was expanded and complemented to cover the full humidity conditions. Fig 7 illustrates the experimental environment.

### Performance evaluation

#### Analysis of the impact of relative humidity on positioning performance

**Fig 8:**
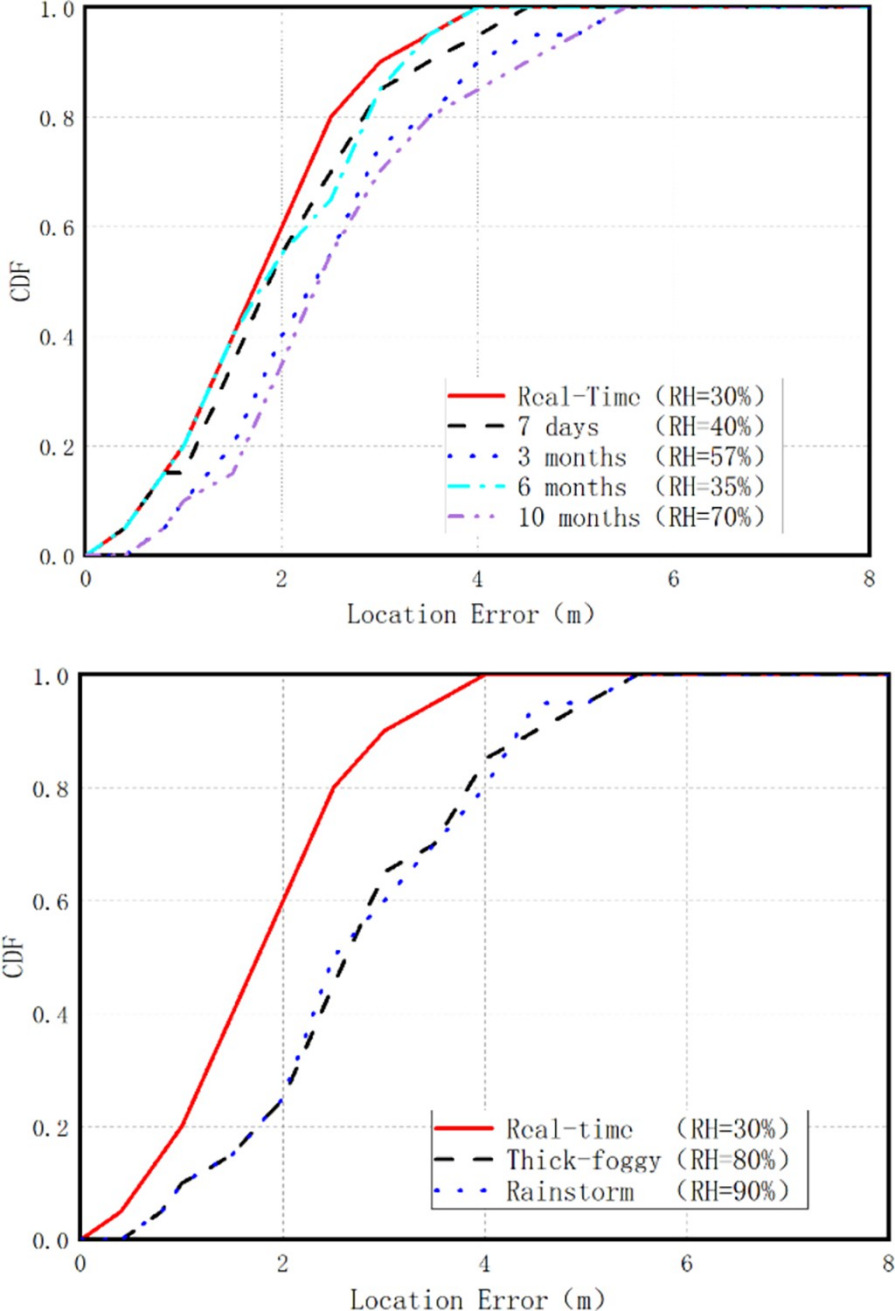
Positioning performance of the fingerprint database under different relative humidity conditions. (a) Positioning performance at different times and under different relative humidity conditions. (b) The positioning performance in dense fog and heavy rain weather.

Experiments are conducted to evaluate positioning in environments with varying levels of relative humidity, testing the impact of relative humidity on positioning performance. We assessed the achievable positioning performance of the unexpanded mixed dual-RF fingerprint database, using fingerprint data from the 7th day, 3rd month, 6th month, and 10th month. The Cumulative Distribution Function (CDF) is depicted in Fig 8(A). As the figure illustrates, the positioning performance of the mixed dual-RF fingerprint database oscillates over time.

The CDF, or Cumulative Distribution Function, is a statistical function commonly used to describe the probability distribution of a real random variable. In this context, the CDF displays the distribution of positioning errors. The y-axis of the CDF graph represents the percentage of positioning errors that are less than or equal to a specific value on the x-axis. For instance, a CDF value of 2.7 meters at the 80% level means that 80% of the positioning errors are less than or equal to 2.7 meters.

When using a real-time constructed fingerprint database, an average positioning accuracy of 2.3 meters can be achieved, and 80% of the positioning errors can be kept under 2.7 meters, thus demonstrating optimal positioning performance results. When utilizing 7-day and 6-month fingerprints, the mixed dual-RF fingerprint database can still achieve average positioning accuracy of 2.6 meters and an 80% positioning error of 2.9 meters. However, when using 3-month and 10-month fingerprints, the positioning performance of the mixed dual-RF fingerprint database significantly declines, with the average positioning error reaching 3.3 meters and 3.7 meters, respectively.

The results indicate that positioning performance fluctuates over the deployment period, and the primary reason for this fluctuation is the difference in relative humidity between the moment of positioning and the real-time constructed fingerprint database. When the difference in relative humidity is large, such as between the real-time fingerprint database (RH = 30%) and the 10-month fingerprint database (RH = 70%), the positioning performance significantly deteriorates. However, when the difference in relative humidity is smaller, such as between the real-time fingerprint database (RH = 30%) and the 7-day (RH = 70%) and 6-month (RH = 35%) databases, the decline in positioning performance is not substantial.

Moreover, to further demonstrate the impact of relative humidity on positioning performance, we selected dense fog and heavy rain conditions for verification. During dense fog, the indoor relative humidity rises to 80%, and during heavy rain, it climbs to 90%. The CDF is depicted in Fig 8(B). As the figure reveals, the positioning performance generally follows the trend that the larger the difference in relative humidity, the more pronounced the decline in positioning performance.

#### Comparison of database expansion performance

The purpose of AeFd model was to achieve the expansion of fingerprint features at a reference point covering all the RH conditions on the premise of obtaining a few fingerprint samples at each reference point. The expanded fingerprint database could have low deviation from the fingerprint database built by real-time collection. Thus, the error precision of RSSI value of finally expanded fingerprint database would serve as an evaluation measure for the AeFd performance.

**Fig 9:**
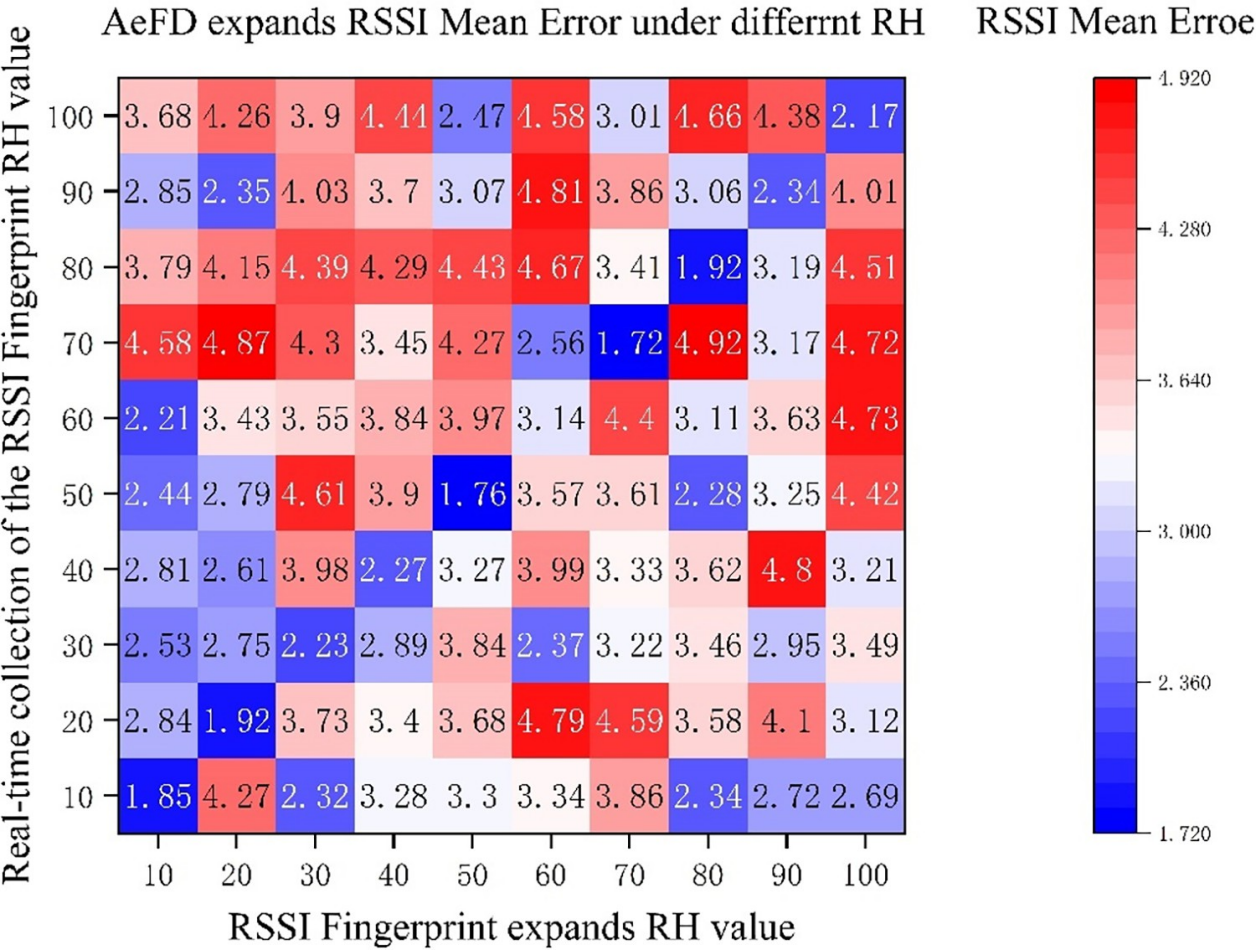
Mean errors between the AeFd expanded RSSI and the real RSSI under different RH.

Fig 9 displays the RSSI prediction errors between the real-time collected fingerprint database and the AeFd-expanded fingerprint database. The data in the figure are the RSSI mean errors between the fingerprint database collected and built under real indoor RHs (as benchmark) and the fingerprint database expanded by AeFd.

As is clear, the AeFd could output accurate predicted RSSI values of fingerprint database at any time under any RH condition. When the humidity value of fingerprint database expanded by AeFd was equal to that during actual collection (upper right diagonal direction in the figure), the RSSI mean error was the smallest, and an expansion effect of RSSI mean error < 2 dbm could be attained. When the humidity value of AeFd-expanded fingerprint database differed from that of fingerprint database during actual collection, the RSSI mean errors were all > 3 dbm, which would exceed 4 dbm especially when such humidity values differed largely.

Meanwhile, the above results also demonstrated presence of a negative correlation between indoor RH and RSSI. That is, when the humidity value during RSSI collection in the online phase differed from that in the construction phase, the RSSI value underwent overall deviation due to the influence of indoor RH, resulting in increased mean error between constructed fingerprint database RSSI and collected RSSI. Moreover, greater differences between the RHs in the fingerprint database collection process and in the online phase led to higher mean errors.

#### Comparison of positioning performance

In this section, positioning performance was used as a measure to verify whether the fingerprint database adaptively expanded by AeFd had better positioning accuracy than the statically built fingerprint database. The widely used classic KNN (K = 5) positioning algorithm was employed to compare the positioning performance between the two fingerprint databases: 1) initial fingerprint database; 2) AeFd model. Noteworthy was that the experimental purpose focused on the improvement in positioning performance achievable by the AeFd model (as compared to the statically built initial fingerprint database), rather than the positioning accuracy attainable by a specific algorithm. Therefore, this section does not optimize the positioning algorithm excessively.

**Fig 10:**
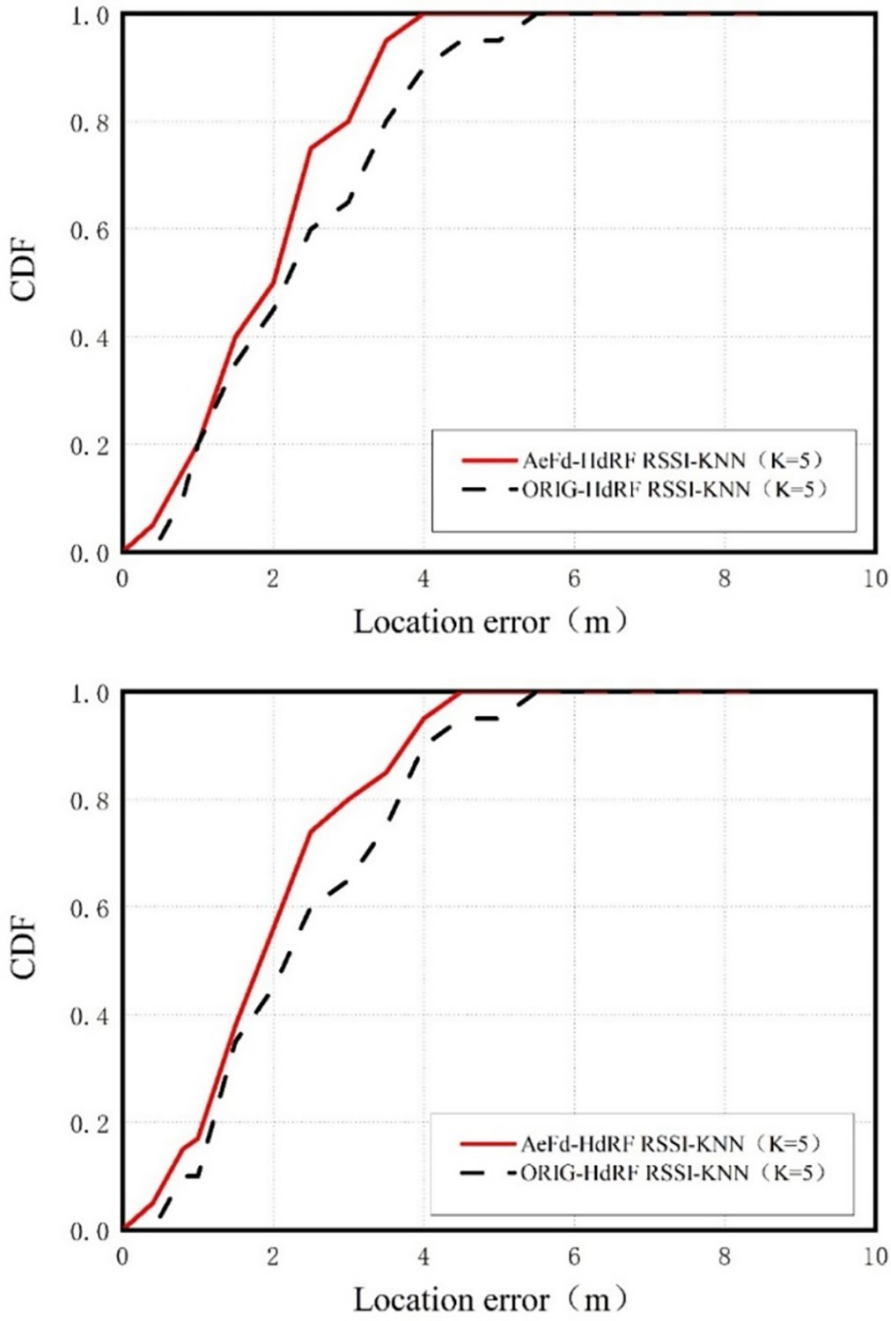
CDF curves of initial and AeFd-expanded fingerprint databases. (a) KNN-based performance on 10th day. (b) KNN-based performance after 10 months.

As is clear from Fig 10(A), when the system was deployed for 10 days, a 5% performance improvement could be achieved by applying the KNN (K = 5) algorithm using the automatically expanded fingerprint database. Fig 10(B) further reveals that when the system ran for over 10 months, an 8% performance improvement could be achieved in the above algorithm using the automatically expanded fingerprint database. According to long-term follow-up observation, the indoor RH fluctuated occasionally in the short-term, while exhibited an overall deviation in the long-term.

Through the above experimentation, it can be found that the primary contribution of the proposed AeFd model to the short-term deployed positioning system lay in selection of fingerprint database corresponding to the specific indoor RH condition, in order to overcome the influence of short-term indoor RH fluctuation on the fingerprint database. Meanwhile, its primary contribution to the long-term deployed positioning system lay in overcoming the overall deviation of indoor RH due to seasonal changes. Therefore, the adaptively expanded fingerprint database can maintain accurate and stable positioning performance for a system running for a long time under gradually changing indoor RHs.

## Summary

RF fingerprinting has emerged as a positioning method possessing significant potential for advancement. However, current WLAN RF fingerprint positioning systems confront numerous pressing challenges. A notably urgent issue is the sharp decline in system positioning accuracy, induced by dynamic alterations in indoor environmental factors, which has garnered escalating attention. This study primarily contributes by thoroughly analyzing how indoor climatic factors potentially influence positioning performance fluctuations and illuminates a previously underappreciated factor impacting the accuracy of fingerprint matching and positioning—indoor relative humidity (RH). Subsequently, to mitigate the impact of this factor on the RF fingerprint Received Signal Strength Indicator (RSSI), an AeFd model, premised on the regression learning algorithm, is introduced. This model facilitates adaptive expansion of fingerprints by employing the RF fingerprint database (amassed under initially known indoor RH conditions) amidst varying indoor climatic and environmental changes, obviating the need for additional hardware deployment or labor costs. Experimental outcomes demonstrate that the proposed method sustains accurate and stable positioning performance for systems operating long-term under gradually shifting indoor RHs.
